# Timing and type of disaster severity data available on Internet following the 2010 Haiti Earthquake

**DOI:** 10.1371/4fb3fd97a2d3c

**Published:** 2012-05-23

**Authors:** Agnes Wefer, Johan Von Schreeb

**Affiliations:** Division of Global Health (IHCAR), Department of Public Health, Karolinska Institutet, Stockholm Sweden

## Abstract

Background To adequately plan relief, adequate information that describe and quantify the severity of a disaster, and estimate the number of affected population, is rapidly needed. However, needs assessments describing the severity of the disaster has been shown to be conducted too late in order to guide the first days relief interventions. The aim of this study was to assess availability of early disaster severity information on Internet during the first seven days following the 2010 Haiti earthquake and assess to which extent the information was consistent with later revelations. Methods We searched the well acknowledged web portal Relief Web for all Haiti postings during the first seven days (12 -18 January 2010) after the earthquake. A form was created to classify and quantify extracted severity variables found in the postings. The results were compiled, analysed and compared with CRED (Centre for Research on the Epidemiology of Disaster) official data made available later. Findings A total of 822 reports were posted where of 15 % provided a numerical estimate of the affected population, while 10% had an estimate on the number of dead. On day four 200 000 dead was reported, which is of the same magnitud compared to later official estimates (CRED data). Not a single report described the data collection method. Conclusions Within a few days of the 2010 Haiti earthquake it was possible to find surprisingly accurate information regarding severity of the earthquake but the available data must be questioned as no method was reported. More specialized and independent needs assessment agencies may improve availability of strategic information in the early onset of a disaster.

## Introduction

On January 12 2010 Haiti and its capital Port au Prince was struck by a devastating earthquake^1^. News about the disaster spread quickly and international relief agencies immediately started deploying relief assistance. To adequately plan relief, information that describe and quantify the severity of the disaster and estimate the number of affected population, is rapidly needed^2^. This type of information combined with pre-disaster context data on vulnerability factors can help estimate the type and quantity of needs as well as what risks the affected population is facing^2^. However, it has been shown that the needs assessments are conducted too late in order to direct the first days interventions^3^ ^4^.

An important information source to rapidly get information about a disaster is the Internet^5^. Information is available within minutes after an earthquake, especially at web portals that focus on disasters and humanitarian assistance. A prime web portal with this focus is ReliefWeb, which is a project of the United Nations Office for Organization for Coordination of Humanitarian Affairs^6^ that is acknowledged as the premier online source of information on sudden onset disasters^7^. In 2009, ReliefWeb had 10 million unique visitors and 4 000 different information providers^8^. A study of information available on ReliefWeb following the 2005 South Asian earthquake showed that reports underestimated the numbers of dead and affected in the first week after the earthquake^9^. New international initiatives aiming at rapidly making information on severity and needs available, have started since then such as the Assessment and Classification of Emergencies project (ACE-project)^6^, the Inter-Agency Standing Committee^10^ and the Assessment Capacities Project^11^. New technologies are more widely available such as increased cell phone and Internet coverage, use of Twitter and other social medias^5^. In the light of this, we wanted to assess to what extent initial severity estimates in the early phase of a major sudden onset disaster is available.

The aim of this study was to assess availability of disaster severity information on Internet during the first seven days following the 2010 Haiti earthquake and assess to which extent the information was consistent with later revelations.

## Methods

ReliefWeb postings are made available by UN branches as well as independent Non Governmental Organizations (NGOs), governments and media channels. Reports, press releases, policy documents and maps are actively searched for by ReliefWeb staff or can be directly submitted from an organization before it is posted on the website^12^.

The word “Haiti” was searched for using ReliefWebs search function and included all postings during the first seven days following the earthquake (12 -18 January 2010). All postings were included in the study.

Two persons read each post and searched for severity indicators. A form was created in EpiData version 3.1^13^ to extract severity indicator variables on the pre-disaster context, the number of affected and dead, needs of the population and the source and methods used. (Table 1) When a range of estimates was provided, a mean value was used. Data was exported to EpiData analysis, version 1.1^14^ to be compiled and basic analyses including cross tabulation for rate and frequency was made. The results on number of affected and dead were chosen to be compared with Haiti official data available at Centre for Research on the Epidemiology of Disaster^15^. CRED data is controlled for quality before being accepted and is to our knowledge the best source for data on disasters. In early 2011 the Haitian government raised the earlier mortality estimate of 222 570 to 316 000^16^. CRED has maintained the earlier number in their database. Results from the Post-Disaster Assessment Report^17^ was used to compare estimates of homeless, internally displaced persons and houses damaged.

**Figure:**
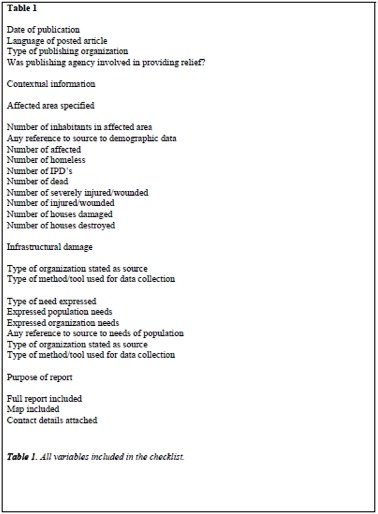
Table 1. All variables included in the checklist.

## Results

A total of 822 reports were posted on ReliefWeb during the first seven days after the earthquake. Of those, 6 % were posted on day one, the 12th of January, the day of the earthquake. The number of posted reports increased the following days. (Figure 1) English was the language in 94.5 % of the reports posted while 4.5 % of the posts were in French and 1 % in Spanish.

**Number of Posts:**
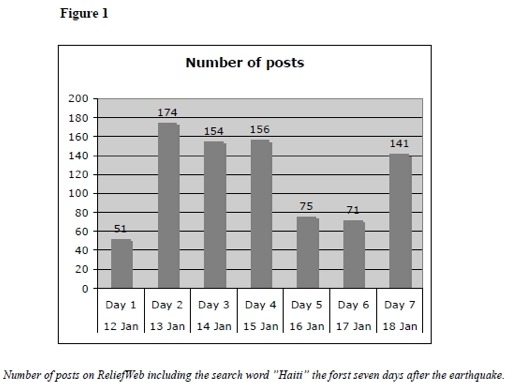
Number of posts on ReliefWeb including the search word "Haiti" the first seven days after the earthquake.

A total of 15 % of the reports provided a numerical estimate of the affected population. The most frequently reported number was 3 million. (Figure 2) The later official number was 3.7 million. A third of all posts providing numbers were posted on day three.

**Number of Affected Reported:**
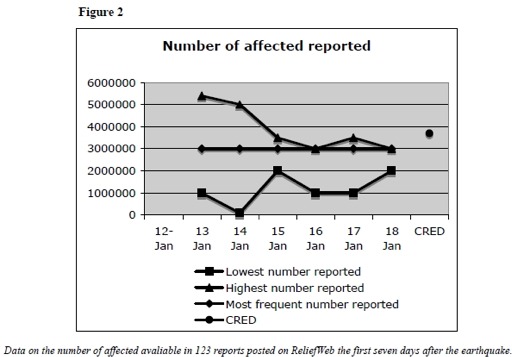
Data on the number of affected available in 123 reports posted on ReliefWeb the first seven days after the earthquake.

A total of 10 % of all the reports included an estimate on the number of dead varying from 40 000 - 200 000. (Figure 3) Estimates on the number of affected, dead, homeless, injured or severely injured were not posted until the day after the quake when the Haiti government claimed that 100 000 people had died. The Haiti Red Cross downgraded this estimate on day three (50 000). On day four, one report citing “local authorities” reported 200 000 dead. This is very close to the official figure (222 570). On the same day, the news agency Reuters-Alertnet posted a quote from the Interior Minister that claimed that 100- 200 000 had died. Also on day four the Chinese news agency, Xinhua, posted that the Haitian President had reported the death to be between 30- 50 000, which is a significantly lower number.

**Number of Dead Reported:**
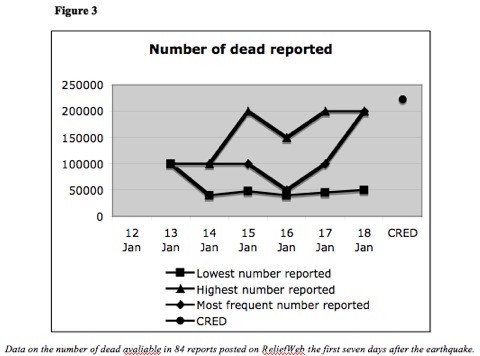
Data on the number of dead available in 84 reports posted on ReliefWeb the first seven days after the earthquake.

Additional severity information including number of wounded, homeless, houses destroyed or internally displaced persons (IDP) was reported in 4 % of the 822 posts. These 22 reports estimated the number of homeless to be between 10 000 to 3 millions. In the PDNA, 1.3 million were declared homeless. Four reports provided a number of IDPs ranging from 30 000 to 1 million compared with 500 000 in the PDNA report. One report estimated the number of houses damaged to 1.8 million, significant higher than the 208 000 in the PDNA data.

Approximately half of the reports expressed a need for the affected population or for the organization that posted the report. The UN was most likely to express population needs while NGOs more often described their own funding resources needs. The type of population needs expressed varied. (Figure 4) The most common request was the combination of shelter, food, water/sanitation, health care as well as search and rescue. The methods used to define the needs were not described in a single report.

**Reported Population Needs:**
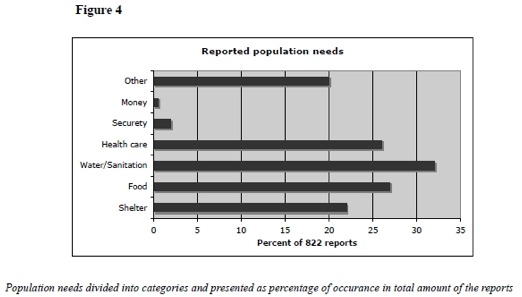
Population needs divided into categories and presented as percentage of occurance in total amounts of reports.

Reference to base line data on the pre-earthquake context was provided in one third of all reports. The fact that Haiti is a low-income country was most commonly mentioned followed by general bad infrastructure, malnutrition, high population density and previous hurricanes/natural disasters. No more detailed information was provided.

A total of 11 % of all postings referred to a data source. Almost 60 % of the posted reports were written by an organization that provided relief themselves. Not a single report described the data collection method.

## Discussions

The result of this study shows that several early estimates of the number of affected and dead were of the same magnitude compare to later official estimates. This can be explained be a number of reasons but it is clear that it is impossible to within a few days estimate the death tolls from such mega disaster. It is striking that numbers of dead and wounded are estimated without any description of how such estimates were done.

On day four, 200 000 were estimated dead, a steep increase, but no method for this is presented. Still today there is controversy of death toll of this earthquake. In January 2011 the official number of dead was upgraded to 316 000 without any further explanations^16^. A yet unreleased report based on a survey indicate a significantly lower death toll of 46 000 – 85 000^18^. There are no reliable methods to early estimate how many people have died, thus early death numbers are highly unreliable while of significant political value. It remains highly debatable whether early death estimates is a useful determinant to categorize the severity of a disaster.

This study has limitations. It only studies one Internet platform. However, we believe that ReliefWeb is the most credible source for rapid detailed post disaster information. Before we started the study we searched for other platforms including GDAC, One World and Alert Net for data and found that information often linked back to ReliefWeb. We concluded that credible institutions post their reports on ReliefWeb. The time period was limited to seven days as we focused on assessing information that may be used by relief agencies responding in this early phase.

Compared to a similar study on postings on ReliefWeb after the 2005 South Asian earthquake^9^ the number of reports posted was the same. (820 vs 822). More than four years later we could not find one single report that systematically tried to quantify and analyze the severity of the disaster and estimate the risks and needs. Much efforts and money have been spent to improve information and improve the tools to monitor disasters. It is noteworthy that there is still no systematic approach available to rapidly assess and analyze the severity of a Sudden Onset Disaster. Initiatives such as The Global Disaster Alert and Coordination System provide near real-time alerts about natural disasters. It is a tool to facilitate response coordination based on computer modeling result but does not attempt to analyze the needs beyond labeling the disasters in green, yellow or red severity levels. At 10.20 PM UTC January 12, 2010, 27 minutes after the earthquake, their automated system generated a red warning^19^. The warning system indicated the severity in terms of population density, intensity of the earthquake and a socioeconomic context^20^.

The fact that a majority of the reports were from agencies involved in providing relief rather than agencies specialized in estimating severity and needs could conflict the information provided. It is surprising that no independent attempt to estimate the needs were posted.

A severity assumption can be rapidly done combining already available pre-disaster data on vulnerability with initial estimates on the severity of the disaster. Several indexes exist for vulnerability at different national and global levels^21^. The initial analyses and assumptions can possibly be done from a distance combining a variety of information and data from different sources. The task to analyze incomplete data and make assumption on severity and projected needs is challenging and requires experience, knowledge and independence. More research is needed to develop robust methods for such analysis.

Another issue is to determine what indicators that best capture the severity of an SOD and define easy methods to collect or estimate them. There is obvious need for independent experts, not directly involved in relief activities, to collect and analyze data and to estimate severity and risks that the population is facing. These experts should have extensive experience of relief work to ensure that they produce relevant results that are useful for operational agencies. More studies are needed to document to what extent disaster severity data is useful for operational agencies. Is the information used? What is the framework for interpreting such data? Is currently available information sufficient to capture critical component of relief planning? Applying systematic tools and methodologies will also allow comparison between Sudden Onset Disasters in different contexts. A promising initiative is the project ACAPS^11^ that is setting up new and independent modalities that within days assess severity after SODs in collaboration with the Interagency Standing Committee Needs assessments Taskforce^22^. We are convinced that better use of new technologies and more specialized needs assessment agencies will help improve availability of strategic information to better plan humanitarian assistance following a SOD.

## Conclusions

Within a few days of the 2010 Haiti earthquake it was possible to find surprisingly good information about the severity of the earthquake. Despite that the estimates were within range of later official data, the reliability of the estimates were questionable as no data collection method was mentioned. Better use of new technologies and more specialized and independent needs assessment agencies could help develop and improve availability of strategic information in the early onset of a disaster.

## Competing Interests

The authors have declared that no competing interests exists.
